# USING TIMEBANK TO IMPROVE VOLUNTEERING AMONG OLDER PEOPLE: A QUASI-EXPERIMENTAL STUDY

**DOI:** 10.1093/geroni/igad104.1301

**Published:** 2023-12-21

**Authors:** Shiyu Lu, Cheryl Chui, Terry Lum

**Affiliations:** City University of Hong Kong, Kowloon, Hong Kong; The University of Hong Kong, Hong Kong, Hong Kong; The University of Hong Kong, Hong Kong, Hong Kong

## Abstract

Timebanking, a system in which people earn time credits through volunteering and exchange earned credits for goods and services, can potentially promote late-life volunteering. However, evidence to support this innovation remains limited. This paper reports the findings of a quasi-experimental study on a timebanking program implemented in six districts in Hong Kong between January 2021 and August 2022. Three districts were assigned as the experimental districts, while others were assigned as the control districts. We recruited 116 older people in the three experimental districts to join the timebank group and 114 older people in three control districts to join the comparison group. Both groups were offered opportunities to volunteer in community elderly centres within their districts (e.g., home visits or phone calls to older people with disabilities). The timebank group could exchange their earned time credit for rewards (e.g., dining coupons and cookies offered by social enterprises), while no reward was offered for the comparison group. Their volunteering hours were recorded using a tailored mobile phone application, and their volunteering intentions were assessed at baseline, during the timebank intervention (7th month), and post-timebank intervention (13th month). After controlling for covariates (e.g., age, years of volunteering experience) using linear mixed models, the timebank group had significantly more volunteering hours (adjusted mean difference [AMD]=1.37, 95% CI: 0.20-2.54, p=0.021) and higher volunteering intention (AMD=0.51, 95% CI 0.29-0.82, p=0.001) than the comparison group. This is the first quasi-experimental study to provide evidence that timebank is a promising and effective approach to promoting late-life volunteering.

